# Real-Time Three-Dimensional Echocardiography to Assess Right Ventricle Function in Patients with Pulmonary Hypertension

**DOI:** 10.1371/journal.pone.0129557

**Published:** 2015-06-15

**Authors:** Yidan Li, Yidan Wang, Zhenguo Zhai, Xiaojuan Guo, Yuanhua Yang, Xiuzhang Lu

**Affiliations:** 1 Department of Echocardiography, Heart Center, Beijing Chao Yang Hospital, Capital Medical University, Beijing, 100020, China; 2 Department of Respiratory Medicine, Beijing Chao Yang Hospital, Capital Medical University, Beijing, 100020, China; 3 Beijing Key Laboratory of Respiratory and Pulmonary Circulation, Beijing Institute of Respiratory Medicine, Capital Medical University, Beijing, China; 4 Department of Radiology, Beijing Chao Yang Hospital, Capital Medical University, Beijing, 100020, China; Temple University, UNITED STATES

## Abstract

**Background:**

The convenience and availability of real-time three-dimensional echocardiography (RT3DE) makes it an attractive candidate for assessing right ventricle function. However, the viability of RT3DE is not conclusive.

**Aim of Study:**

This study aims to evaluate RT3DE relative to cardiac magnetic resonance and 2-dimensional echocardiography (2DE) for measuring right ventricular systolic function in patients with pulmonary hypertension.

**Methods:**

Patients with pulmonary hypertension (n = 23) underwent cardiac magnetic resonance, 2DE, and RT3DE. Specifically, 2DE was used to measure the right ventricular index of myocardial performance (RIMP), fractional area change, tricuspid annular plane systolic excursion (TAPSE), and tissue Doppler-derived tricuspid annular systolic velocity (S′). Cardiac magnetic resonance and RT3DE were used to measure right ventricular end-diastolic volume (RVEDV) and end-systolic volume (RVESV). The right ventricular ejection fraction (RVEF) was calculated.

**Results:**

Regarding the measurements taken by 2DE, RVEF positively correlated with fractional area change (*r* = 0.595, *P* = 0.003) and S′(*r* = 0.489, *P* = 0.018), and negatively correlated with RIMP (*r* = −0.745, *P* = 0.000). There was no association between RVEF and TAPSE (*r* = −0.029, *P* = 0.896). There existed a close correlation between the values of RVEDV, RVESV, and RVEF as measured by RT3DE and CMR respectively (*P*<0.001); Bland-Altmanan analyses showed good agreement between them.

**Conclusion:**

RT3DE was a viable method for noninvasive, accurate assessment of right ventricular systolic function in patients with pulmonary hypertension.

## Introduction

Most patients with pulmonary hypertension have marked right ventricle (RV) dysfunction. In many pathological conditions, RV function is an important predictor of prognosis, as it is strongly associated with clinical outcomes, disease severity, and patient health-related quality of life. Thus, accurate assessment of RV structure and function is crucial to the management of patients with pulmonary hypertension.

Two-dimensional (2D) echocardiography (2DE) is the most commonly used clinical imaging method for functional evaluation of the right ventricle. With 2DE, the complex geometry structure of the RV, with both a crescent shape and an outspread inflow and outflow tract requires several scan planes for the estimation of RV size and function. However, M-Mode and tissue Doppler imaging of the free lateral wall of the RV are measured in one plane clinically, and are used as surrogates for RV function. Hence, current echocardiographic techniques are not suitable for calculating right ventricular volumes and function accurately with a simple algorithm [1]. Real-time three-dimensional (3D) echocardiography (RT3DE) is able to display the 3D anatomy of the RV; simultaneously depicting both longitudinal and transverse movements [2].This feature makes it superior to conventional two-dimensional methods in RV function assessment. However, the feasibility and accuracy of RT3DE has not been fully studied.

Cardiac magnetic resonance imaging (CMR) provides multilane, high-contrast, high-resolution images that can be analyzed without reliance on geometric assumptions for assessing the right ventricle. CMR is used to determine the RV end-diastolic and end-systolic volumes, to calculate RV ejection fraction (RVEF) [3]. In this study, to determine the feasibility and accuracy of RT3DE for measuring the RV function of patients with pulmonary hypertension, RT3DE was evaluated relative to CMR for measuring right ventricular global volume and systolic function.

## Materials and Methods

### Study patients

We studied 23 consecutive adult patients with pulmonary hypertension at Beijing Chaoyang Hospital between October 2013 and July 2014, who were naive to pulmonary hypertension management. The cohort consisted of 13 patients with chronic thromboembolic pulmonary hypertension, 8 with idiopathic pulmonary arterial hypertension, and 2 with connective tissue diseases associated with pulmonary arterial hypertension. All these patients underwent both RT3DE and CMR to assess right ventricular function, at the basis of 2DE in accordance with American Society of Echocardiography (ASE) recommendations [4]. The study followed the guidelines of the Helsinki Declaration and was approved by the Ethics Committees of Beijing Chaoyang Hospital. Written informed consent was obtained from all participants. After obtaining approval from the Institutional Review Board, the patients’ medical records were collected for the evaluation of patient demographics, clinical characteristics, and right-heart catheterization data.

### 2DE

2DE was performed with a Philips iE33 ultrasound system (Philips, The Netherlands), and images were analyzed offline after the procedures. The tricuspid annular plane systolic excursion (TAPSE) was acquired by M-mode. The M-mode cursor was placed through the lateral aspect of the tricuspid annulus, such that the annulus moved along the M-mode cursor. The systolic displacement was measured from end-diastole to end-systole. The tricuspid annular systolic velocity (S′) was measured via tissue Doppler imaging in the apical four-chamber view. The isovolumic acceleration of the RV was calculated as the peak isovolumic myocardial velocity divided by the time to peak velocity, as measured by tissue Doppler imaging at the lateral tricuspid annulus. The RV diastolic area and RV systolic were obtained from the two-dimensional apical four-chamber view. The right ventricular fractional area change (RVFAC) was calculated as: RVFAC = (RV diastolic area–RV systolic area)/RV diastolic area×100%.The right ventricular index of myocardial performance (RIMP) was calculated as the ratio of the isovolumic time to the ejecting time, which were measured in the same pulsed tissue Doppler imaging. The isovolumic time was calculated by subtracting the ejecting time from the tricuspid closure time. The ratio of the RV transverse diameter to the left ventricular transverse diameter was measured at the base in end-diastole using the apical four-chamber view.

### RT3DE

RT3DE with full volume and harmonic imaging was recorded over 4–7 cardiac cycles with a matrix-array transducer. All recordings were electrocardiogram-gated and performed with a breath-hold technique. The images were gathered with an x3-1(iE33, Philips Health care) and a 4Z1C matrix-array transducer (Siemens Acuson SC2000).The apical view was used for recording although with a more off-axis approach to include the entire RV. Depth, sector size, angle, and focus were adjusted to focus on the region of interest in the RV. Two to four recordings were acquired for the RV. The datasets were saved in a digital format to a workstation connected to a TomTec server (TomTec Imaging Systems, Unterschleissheim, Germany) for further analyses. RV data were interpreted with 4D RV-Function (TomTec Imaging Systems) software. Before tracing, end-systole and end-diastole volumes were defined as the smallest and largest cavities, respectively. The endocardium was traced manually in end-systole and end-diastole in the 4-chamber, short-axis and coronal views. The endocardial border was detected semi-automatically, over a full cardiac cycle. Manual correction was performed to optimize the endocardial border delineation in all patients.

### CMR

CMR was performed with a 3.0 Tesla magnetic resonance scanner (TimTrio; Siemens, Erlangen, Germany) using a four-channel cardiac phased-array surface coil for data acquisition. All CMR data were transferred to a workstation (SynogoMMWP VE30A; Siemens, Berlin, Germany), and analyzed with validated software (Argus, ventricular function; Siemens Medical Systems, Erlangen, Germany).CMR was performed with the gradient-echo pulse sequence (True FISP; repetition time/echo time, 48.0/1.6ms; flip angle, 50°; matrix, 256×256 pixels; field of view, 340mm; section thickness, 6mm) using retrospective electrocardiogram triggering during breath holding. Twenty-five frames were obtained for each cardiac cycle. The right and left ventricles were semi-automatically segmented by an experienced radiologist who identified the endocardial and epicardial boundaries. Function parameters, including the RV end-diastolic volume (RVEDV), RV end-systolic volume (RVESV), RV ejection fraction (RVEF), RV stroke volume, and RV cardiac output were automatically calculated by the software. Similar cardiovascular parameters were obtained for the left ventricle using the same methods.

### Statistical analyses

Continuous variables are expressed as mean± standard deviation. Pearson’s correlation coefficients were calculated for analysis of the associations among the parameters of interest. A Bland-Altman plot was used to show differences and limits of agreement in the measurements of the RV EDV index, RV ESV index, and RVEF between RT3DE and CMR. A *P*-value less than 0.05 were considered statistically significant. The statistical software SPSS (version 17.0 for Windows; SPSS, Chicago, IL, USA) was used for statistical analysis and graphic presentation.

## Results

### Clinical features

The study population consisted of 23 patients in end-stage of pulmonary hypertension; 10 men and 13 women, aged of 51.6 ± 14.8 years, and an average body mass index of 24.36 ± 2.67 kg/m^2^ (Table 1). The hemodynamic parameters were measured by right heart catheterization. The mean pulmonary artery pressure was 57.35 ± 17.82 mmHg, and the pulmonary vascular resistance was 1378.50 ± 830.12dyn S cm^-5^.

**Table 1:** Patients characteristics (n = 23).

Number of patients		23
Age, y		51.6±14.8
Gender, n(%)	Men	10(43%)
	Women	13(57%)
Body mass index, kg/m^2^		24.36±2.67
Heart rate, beats/min		83±14(61–115)
Blood pressure, mmHg	Systolic	114±14(90–150)
	Diastolic	74±8(56–90)
Six-minute walk distance, m		311.60±146.58(30–501)
Mean pulmonary arterial pressure, mmHg		57.35±17.82(32–115)
Pulmonary capillary wedge pressure, mmHg		8.00±2.13(5–13)
Pulmonary vascular resistance, dyn S cm^-5^		1378.50±830.12(418–3403)
Central venous pressure, mmHg		8.21±5.84(4–20)

### RV measurement

The echocardiography and CMR of the RV were conducted on the same day within 24 hours (Table 2). Results of the 2DE showed a TAPSE of 13.94 ± 2.23mm, S′of 9.79 ± 1.02cm/s, fractional area change of 32.05 ± 8.27%, and RIMP of 0.81 ± 0.22. RT3DE examination showed RVEDV of 128.66 ± 35.72mL, RVESV of 81.70 ± 24.36mL, and RVEF of 36.54 ± 6.10%. CMR examination showed RVEDV of 138.39 ± 39.20mL, RVESV of 85.11 ± 28.19mL, RVEF of 38.91 ± 8.15%.

**Table 2:** Echocardiography and CMR data.

			Value
Echocardiography	2DE	TAPSE, mm	13.94±2.23
		S′, cm/s	9.79±1.02
		RVFAC, %	32.05±8.27
		RVMPI	0.81±0.22
	RT3DE	RVEDV, mL	128.66±35.72
		RVESV, mL	81.70±24.36
		RVEF, %	36.54±6.10
CMR		RVEDV, mL	138.39±39.20
		RVESV, mL	85.11±28.19
		RVEF, %	38.91±8.15

RVMPI: right ventricle myocardial performance index.

### Correlation analysis of RT3DE-derived RVEF and 2DE-derived parameters

**Fig 1:**
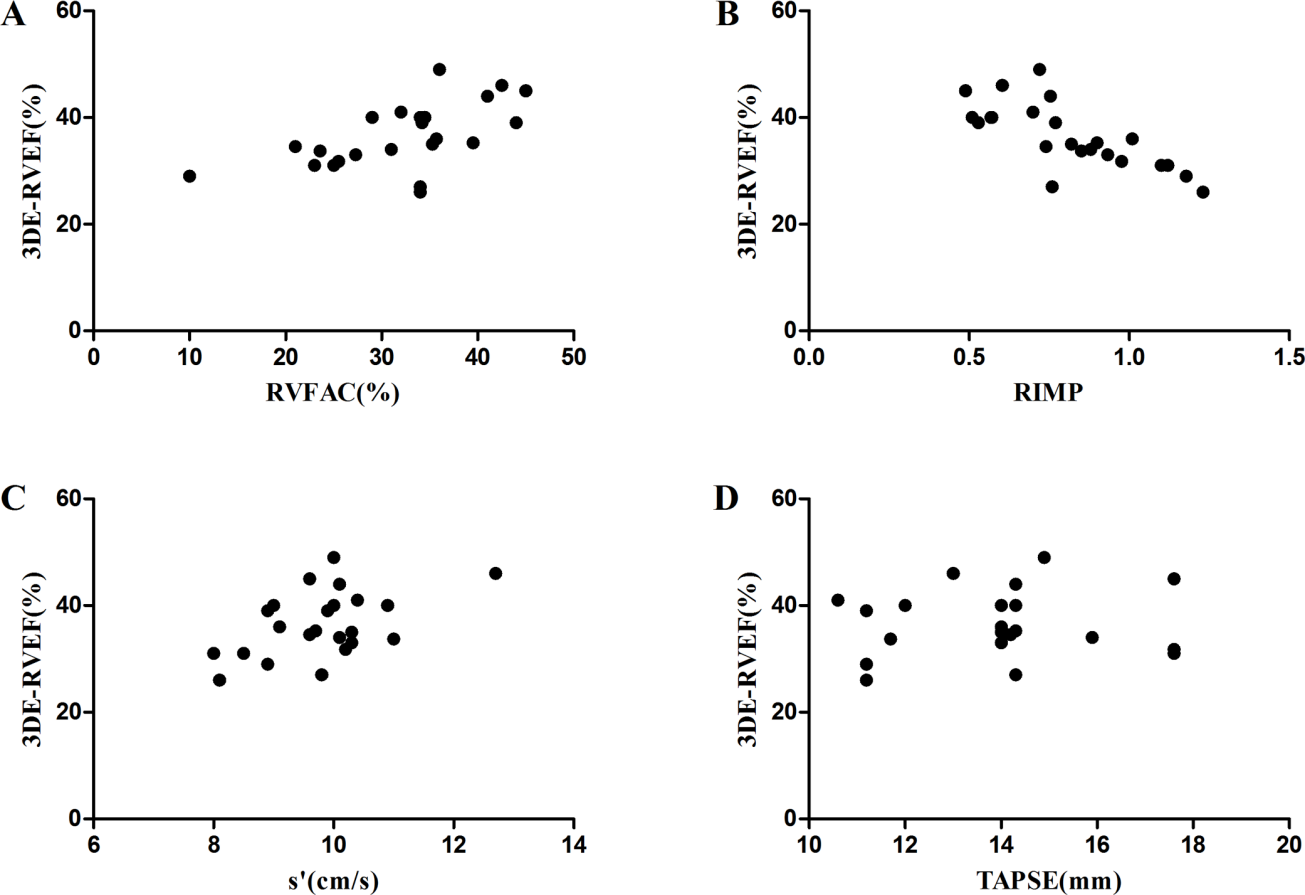
Correlation between 2DE parameters and RT3DE-derived RVEF. (A) Correlation between RVFAC and RT3DE-derived RVEF. (B) Correlation between RIMP and RT3DE-derived RVEF. (C) Correlation between S′and RT3DE-derived RVEF. (D) Correlation between TAPSE and RT3DE-derived RVEF.

The RT3DE-derived RVEF significantly correlated with S′ (*r* = 0.489, *P* = 0.018), RVFAC (*r* = 0.595, *P* = 0.003), and RV myocardial performance index (*r* = –0.745, *P* = 0.000; Fig 1). However, there was no correlation between RT3DE-derived RVEF and TAPSE (*r* = –0.029; *P* = 0.896).

### Correlation analysis of RT3DE-derived and CMR-derived parameters

**Fig 2:**
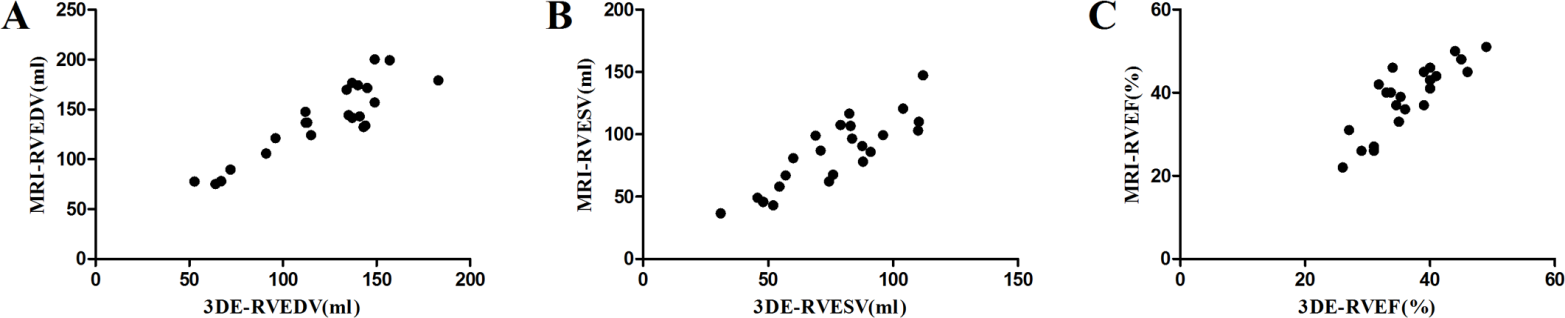
Correlation between RT3DE-derived and CMR-derived RV parameters. (A) Correlation of RVEDV values obtained via RT3DE and CMR. (B) Correlation between RVESV values measured via RT3DE and CMR. (C) Correlation between RVEF values measured by RT3DE and CMR.

RT3DE-derived RVEDV, RVESV, and RVEF also correlated well with CMR-derived RVEDV, RVESV and RVEF, respectively (Table 3 and Fig 2). The coefficient *r* coefficient for the correlation of RVEDVs in the RT3DE and CMR measurements was 0.891(*P* = 0.000), of RVESVs was 0.859(*P* = 0.000), and of RVEFs was 0.833(*P* = 0.000).

**Table 3:** Correlations between RT3DE-derived and CMR-derived RV parameters.

	RVEDV	RVESV	RVEF
*r*	0.891	0.859	0.833
*P*	0.000	0.000	0.000

### Agreement analysis of RT3DE-derived and CMR-derived parameters

**Fig 3:**
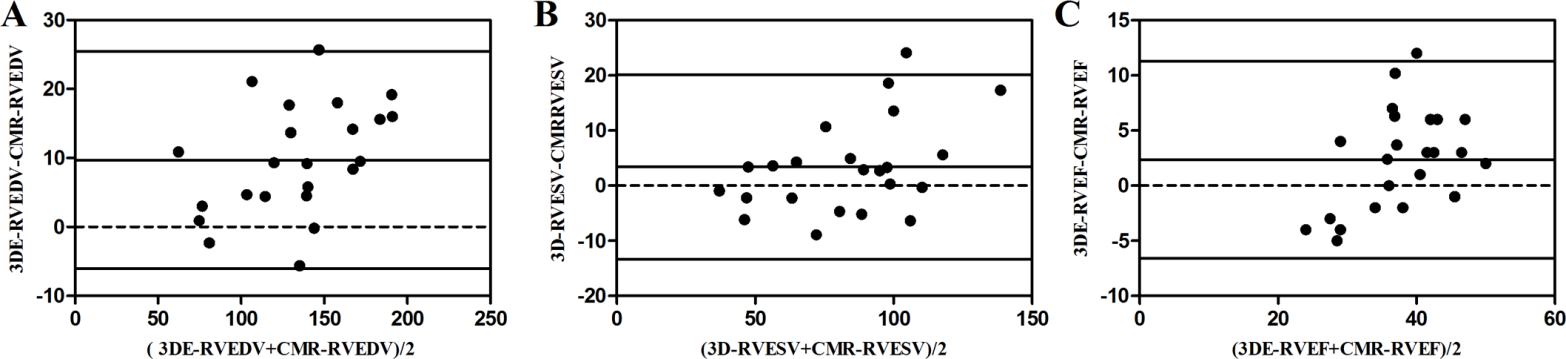
Bland-Altman analysis of parameters measured by RT3DE and CMR. (A) Bland-Altman analyses of RT3DE and CMR measurements of RVEDV. (B) Bland-Altman analysis of RT3DE and CMR measurements of RVESV. (C) Bland-Altman analysis of RT3DE and CMR measurements of RVEF.

A Bland-Altman plot showed small mean differences and narrow limits of agreement between RT3DE and CMR in the measurements of the RVEDV and RVESV indices, and RVEF (Fig 3). For RVEDVs, the bias was 9.7, 95% limits of agreement were −6.0 to 25.5, and all measurements fell within the limits. For RVESVs, the bias was3.4, 95% limits of agreement was −13.3 to 20.1 and only one in 23 measurements fell outside the limits. For RVEFs, the bias was 2.4, 95% limits of agreement was −6.6 to 11.3, and only one in 23 measurements fell outside the limits.

## Discussion

Pulmonary hypertension is a life-threatening disease characterized by chronic elevation of mean pulmonary arterial pressure. Because of the lack of signs and symptoms, a majority of patients in the end-stage of disease are not on treatment. Early identification of pulmonary hypertension and accurate evaluation of RV function is crucial for clinical treatments [5], as RV function is strongly associated with clinical outcomes, severity of disease, patient health-related quality of life, and prognosis [6].

Increased understanding of the mechanism underlying pulmonary hypertension has highlighted the importance of right heart function in patients with this condition [5]. However, the complexity of RV morphology limits the precise measurement and evaluation of RV function. Currently, CMR is the most accurate method for measuring RV structure; therefore, it is considered the reference method for any new imaging method. The measurement of RV volume by CMR uses the disk summation method based on Simpson’s rule, which depends on steady-state free precession cine cardiac images [7]. Although CMR is the gold standard for measuring RV volume, its use for monitoring RV function is limited by the expense, the duration of the procedure, and patients with implanted devices. In addition, CMR requires a lengthy breath hold, which can cause suffering for patients with heart failure. In contrast, echocardiography is convenient, inexpensive, and tolerable for repeated examinations, even in patients with implantable devices. Some patients with severe pulmonary hypertension have intravenous infusion pump devices fitted for continuous administration of medication, possibly rendering them contraindicated for CMR. In these cases, RT3DE may be an ideal alternative for evaluating RV volume and function [8–9]. The 2010 ASE guidelines for echocardiographic assessment of the right heart in adults recommend that conventional echocardiographic diagnosis should include several indices that can be easily measured with high repeatability and reproducibility. These indices include TAPSE, S′, fractional area change, and RIMP. TAPSE represents the longitudinal function of the right ventricle. It is simple, reproducible, and less dependent on image quality, and does not require sophisticated equipment or prolonged image analysis. Integration of S′ by pulsed tissue Doppler is a simple and reproducible measurement to assess basal function of the RV free wall. RIMP is a global estimate of both systolic and diastolic function of the RV, which avoids the geometric assumptions and limitations of complex RV geometry. 2D fractional area change is a recommended method in quantitatively estimating RV function. Measuring more than one index of RV function is suggested, as this provides a more reliable assessment of right heart function [4]. Because of the heterogeneity of methods and the numerous geometric assumptions, 2D image-derived estimation of RVEF is not recommended. In contrast, RV volumes and ejection fraction can be accurately measured by 3D echocardiography using validated real-time 3D algorithms. In the present study, we analyzed the correlation between RT3DE-derived RVEF and 2DE-derived TAPSE, S′, fractional area change, and RIMP in pulmonary hypertension patients. We found that RVEF correlated with fractional area change, RIMP and S′, but not with TAPSE. One of the main disadvantages of evaluation of RV function from one single plane is that it only provides a limited perspective. For instance, if tricuspid regurgitation or longitudinal shortening of different segmental function is not displayed on the four chamber view, there will be a poor correlation between TAPSE and ejection fraction [10–11]. Under the condition of volume or pressure overload, even if short axis contour analysis indicates dilatation and dysfunction of the right ventricle, longitudinal analysis will show poor correlation between TAPSE and ejection fraction [12–13].

RT3DE is a new imaging method for noninvasive evaluation of cardiac function that has been applied clinically [14–16].In the present study, the RV volumes were underestimated by RT3DE compared with CMR, which is similar to prior studies [17–18]. The main reason for the underestimation of RV volume is that 3D echocardiography cannot fully display the RV outflow tract, especially when the right ventricle is severely dilated. Meanwhile, it is a little difficult for 3D echocardiography to identify the anterior wall of the right ventricle. Because the underestimation of the end-diastolic and systolic volumes was coincident and self-corrected, therefore calculations of the RVEF did not differ between the 2 methods. Moreover, to avoid the acoustic shadow of the sternum or lung tissue, the RV outflow tract can not be fully displayed when 3D echocardiography is used to acquire RV volume data, and obesity can cause ultrasonic penetrance problems because of the substantial amount of tissue in front of the heart. On the other hand, underweight patients can make it difficult to get sufficient probe-skin contact between the ribs. One study reported that 3D echocardiography was feasible for imaging the RV in a large proportion (85%) of adult patients with unselected pathology [1]. For all this, RT3DE is an ideal method for evaluation of RV function in patients with pulmonary hypertension, and can provide guidance for the evaluation of clinical prognosis and selection of treatment.

Our study found that RT3DE was a viable method for noninvasive, accurate assessment of right ventricular systolic function in patients with pulmonary hypertension. However, our study also has limitation. The sample size of the study is small, which needs to be proved in the further study.
